# *In Vitro* Antiplasmodial Activity of Phospholipases A_2_ and a Phospholipase Homologue Isolated from the Venom of the Snake *Bothrops asper*

**DOI:** 10.3390/toxins4121500

**Published:** 2012-12-14

**Authors:** Juan Carlos Quintana Castillo, Leidy Johana Vargas, Cesar Segura, José María Gutiérrez, Juan Carlos Alarcón Pérez

**Affiliations:** 1 Center for the Study of Biological Systems, Cooperative University of Colombia, Medellín 050012, Colombia; 2 Ophidism/Escorpionism Program, University of Antioquia, Medellín 050012, Colombia; E-Mails: johana2104@gmail.com (L.J.V.); juan.alarcon@siu.udea.edu.co (J.C.A.P.); 3 Malaria Group, School of Medicine, University of Antioquia, Medellín 050012, Colombia; E-Mail: cesar.segura@siu.udea.edu.co; 4 Clodomiro Picado Institute, School of Microbiology, University of Costa Rica, San José 1000, Costa Rica; E-Mail: jose.gutierrez@ucr.ac.cr

**Keywords:** snake venom, *Plasmodium falciparum*, *Bothrops asper*, phospholipase A_2_, enzymatic activity, phospholipase A_2_ homologue

## Abstract

The antimicrobial and antiparasite activity of phospholipase A_2_ (PLA_2_) from snakes and bees has been extensively explored. We studied the antiplasmodial effect of the whole venom of the snake *Bothrops asper* and of two fractions purified by ion-exchange chromatography: one containing catalytically-active phospholipases A_2_ (PLA_2_) (fraction V) and another containing a PLA_2_ homologue devoid of enzymatic activity (fraction VI). The antiplasmodial effect was assessed on *in vitro* cultures of *Plasmodium falciparum*. The whole venom of *B. asper*, as well as its fractions V and VI, were active against the parasite at 0.13 ± 0.01 µg/mL, 1.42 ± 0.56 µg/mL and 22.89 ± 1.22 µg/mL, respectively. Differences in the cytotoxic activity on peripheral blood mononuclear cells between the whole venom and fractions V and VI were observed, fraction V showing higher toxicity than total venom and fraction VI. Regarding toxicity in mice, the whole venom showed the highest lethal effect in comparison to fractions V and VI. These results suggest that *B. asper* PLA_2_ and its homologue have antiplasmodial potential.

## 1. Introduction

Malaria is responsible for approximately 1.5 million deaths every year in the world. Over 85% of them occur in Africa, with *Plasmodium falciparum* as the leading species involved in mortality [1,2]. The 2010 WHO report confirmed almost 1 million deaths during the previous year [3]. Malaria is caused by parasites of the genus *Plasmodium* and is a public health problem in tropical and sub-tropical regions of the world. The most widely used treatment of the clinical syndrome includes artemisinin-based combined therapies [3]. High rates of antimalarial treatment failure have led to the investigation of possible therapeutic alternatives, among which toxins and poisons of animal and plant extracts are included [4,5,6,7,8,9].

The viperid snake species *Bothrops asper* is widely distributed throughout America, from southern Mexico to northern Ecuador [10]. Its venom is a complex mixture of peptides, enzymes and toxins, including metalloproteases (41%–44%), phospholipases A_2_ (PLA_2_) (29%–45%), serine proteases (4%–18%), L-amino acid oxidases (5%–59%), disintegrins (1%–2%) C-type lectin-like proteins (0.5%) and cysteine-rich secretory proteins (CRISP) (0.1%) [11], which are responsible for the toxicity of the venom and result in the complex pathophysiology provoked by these envenomations, characterized by coagulopathy, hemorrhage, blistering, edema, nephrotoxicity, shock and myotoxicity [12].

The PLA_2_ (E.C 3.1.1.4) superfamily includes enzymes that hydrolyze phospholipids, specifically the *sn*-2 ester bond, to produce fatty acids and lysophospholipids. Secreted PLA_2_s (sPLA_2_) share several characteristics: low molecular mass (13–18 kDa), numerous disulfide bonds, histidyl and aspartyl catalytic residues and a highly conserved calcium (Ca^2+^) binding region [13,14]. PLA_2_s from snake venom exhibit a variety of pharmacological/toxicological activities, such as myotoxicity, neurotoxicity, anticoagulant activity, edema-forming activity, cardiotoxicity, antibacterial activity, antiparasite effect and anti-aggregation activity on platelets, among others [15,16,17,18,19,20,21,22,23,24,25].

Based on the already described antimicrobial and anti-parasitic activity of PLA_2_ [17,25,26,27,28] from snake venoms, the antimalarial potential of the venom of *B. asper* and PLA_2_s from this venom were explored. Two PLA_2_s from the whole venom were purified and characterized, and their *in vitro* antiplasmodial activity against *P. falciparum* was investigated. Cytotoxicity on peripheral blood mononuclear cells (PBMC) and acute toxicity in mice were also evaluated. Results indicate that catalytically-active and inactive PLA_2_s isolated from *B. asper* venom are cytotoxic against *P. falciparum* and, thus have the potential as antimalarials.

## 2. Results

### 2.1. Isolation of Phospholipase A_2_ Fractions

**Figure 1:**
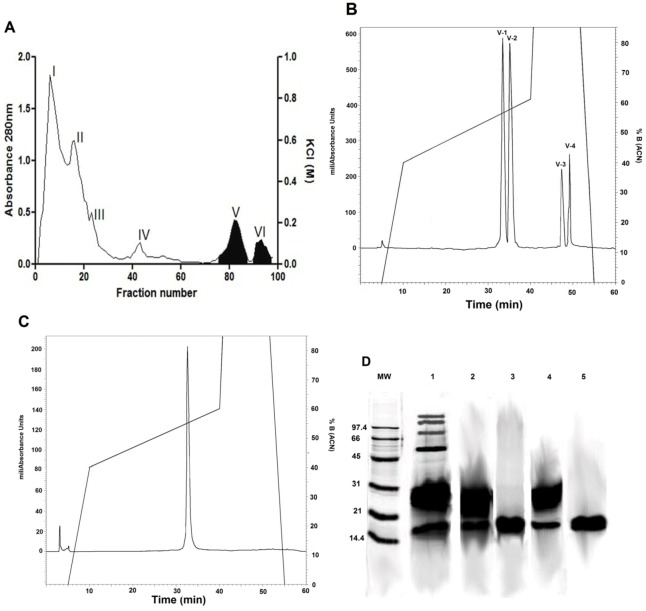
(**A**) Chromatographic elution profile on CM Sephadex C-25 at 280 nm from the venom of *B. asper*; fractions V and VI (shaded) were further characterized; (**B**) Elution profile on RP-HPLC on a C_18_ column of fraction V; (**C**) Elution profile on RP-HPLC on a C_18_ column of fraction VI; (**D**) SDS-PAGE (12%) separation of venom and fractions: *MW*, molecular weight markers; lane 1, crude venom; lane 2, fraction V under non-reducing conditions; lane 3, fraction V under reducing conditions; lane 4, fraction VI under non-reducing conditions; lane 5, fraction VI under reducing conditions.

Six fractions obtained by fractionating *B. asper* venom on ion exchange chromatography on CM-Sephadex C-25 were evaluated for PLA_2_ activity. It was found that fraction V was the only positive fraction for PLA_2_ activity (see Figure 1A). However, fraction VI, corresponding to a PLA_2_ homologue devoid of enzymatic activity (see Section 3.2), was also analyzed for antiplasmodial activity to determine the possibility of catalytically-independent actions. Fractions V and VI were subjected to further separation by RP-HPLC on a C_18_ column. This separation revealed that fraction V had four subfractions (see Figure 1B,C), only one of which (V-4) showed PLA_2_ activity, whereas fraction VI showed only one peak. These two fractions were used to assess antiplasmodial activity.

### 2.2. Indirect Hemolytic Activity

**Figure 2:**
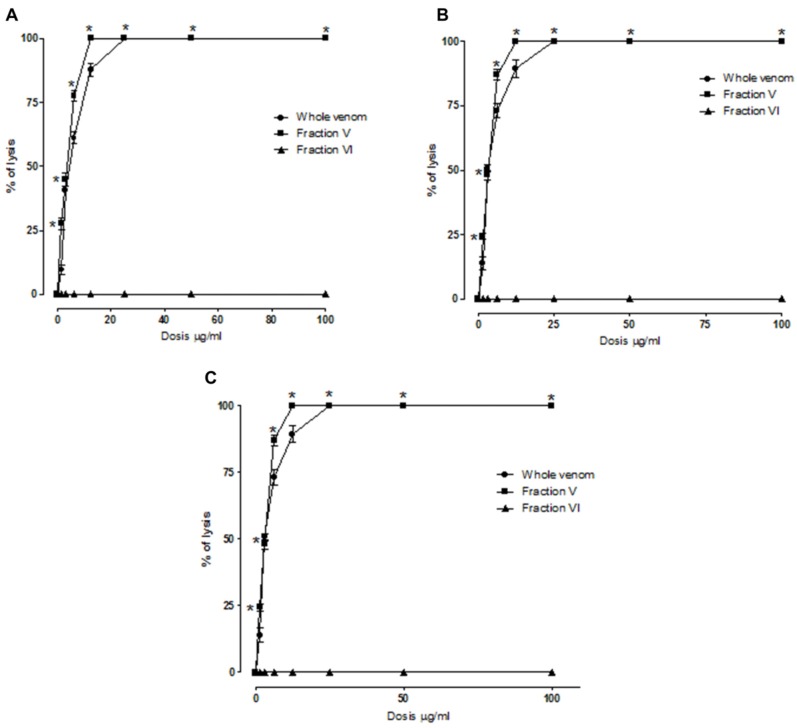
Indirect hemolytic activity in solution of venom and fractions V and VI. Analysis on erythrocyte suspensions containing (**A**) egg yolk; (**B**) inactivated human plasma and (**C**) inactivated human serum. * *p* ≤ 0.05.

Fraction V had a minimal indirect hemolytic dose of 1.35 µg, while fraction VI showed no such activity. The PLA_2_ isolated by HPLC from fraction V showed a minimum indirect hemolytic dose of 0.82 µg, while the peak obtained by HPLC separation of fraction VI lacked activity (data not shown). The hemolysis test with different substrates (egg yolk, plasma or human serum) yielded similar results in all assays. When indirect hemolytic activity was determined in solution, 100% hemolysis was observed using concentrations of 25 µg/mL for whole venom and 12.5 µg/mL for fraction V, whereas fraction VI lacked PLA_2_ activity in all tests (see Figure 2).

### 2.3. Antiplasmodial Activity of the Venom, Fractions and Purified PLA_2_s

Both venom and fractions V and VI exhibit antiplasmodial activity on the FCB1 strain of *P. falciparum*, with fraction V being more active than fraction VI (see Table 1). On the other hand, the venom was more active than the two fractions evaluated. Guillaume *et al.* showed that removal of phospholipids from cultures of *P. falciparum* reduced the antiplasmodial activity of PLA_2_ [27], confirming the crucial role of PLA_2_ enzymatic activity to control the growth of parasites in this test. Our data demonstrate the antimalarial efficacy of fraction with PLA_2_ activity. However, a PLA_2_ homologue devoid of enzymatic activity also resulted in restriction of *P. falciparum* multiplication, confirming a catalytically-independent antiplasmodial activity. This effect could be due to the perturbing action exerted by the PLA_2_ homologue in the plasma membrane, thus resulting in an increase in permeability [29]. It has been shown that the *C*-terminal region of these PLA_2_ homologues is responsible for this catalytically-independent membrane perturbation, as demonstrated in bacteria [16,30,31], being, therefore, a different mechanism from the one described for other PLA_2_s [26,27]. 

**Table 1:** Antimalarial activity, cytotoxic activity on peripheral blood mononuclear cells and acute toxicity of *B. asper* venom and isolated PLA_2_s. ND: not determined. ^£^ No deaths were recorded at this dose. ^€^ *p* ≤ 0.05 when compared with the other treatments.

Compound	Antimalarial activity IC_50_ (µg/mL)	LD_50_ (µg/kg)	Cytotoxicity CC_50_ (µg/mL)
*B. asper* venom	0.13 ± 0.01 ^€^	3566 (2561 to 3693)	38.46 ± 0.95 ^Ω^
Fraction V	1.42 ± 0.56 ^€^	^£^ > 15000	26.98 ± 0.51 ^Ω^
Fraction VI	22.89 ± 1.22 ^€^	^£^ > 15000	67.43 ± 1.03 ^Ω^
CQ *	323.35 ± 6.97	ND	ND

* CQ: chloroquine. These results are expressed in nM concentration; CC_50_: Dose that induces 50% cytotoxicity in peripheral blood mononuclear cells. Results are expressed as mean ± S.E.M.; ^Ω^ *p* ≤ 0.05 when compared with the other treatments.

The changes observed in the intraerythrocytic development of *Plasmodium* indicate that structural changes occur, as well as modifications in membrane functions in parasitized red blood cells. In addition, increments and changes in the permeability of the membrane have been described, together with the appearance of new parasite-derived proteins and changes in the composition of membrane lipids [32,33]. The observed increased permeability could also be responsible for the PLA_2_ activity on the parasite, as demonstrated by Moll *et al*., who noted that in the absence of serum in the culture *in vitro*, PLA_2_ lysed parasitized cells [34]. This increase in membrane permeability could also enhance the antimalarial activity of the PLA_2_ homologue observed in our experiments.

### 2.4. SDS-PAGE

Electrophoresis showed that proteins of fractions V and VI (lanes 2 and 4, respectively) had molecular weights ranging from 25 kDa and 14 kDa, when fractions were separated in non-reducing conditions, thus evidencing the presence of monomers and dimers, whereas only bands of around 14 kDa were observed (lanes 3 and 5 in Figure 1D, respectively) when these fractions were subjected to reducing conditions, thus corresponding to PLA_2_ monomers (Figure 1D).

### 2.5. Mass Spectrometry and Identification of the Protein

We determined the molecular mass of each of the fractions obtained by RP-HPLC: Fraction V (fractions V-1, V-2, V-3 and V-4) and VI. Mass spectrometric analysis showed that V-1 was of 13786.9 Da, V-2 was of 13950.1 Da, V-3 was of 13972.4 Da, V-4 was of 13974.6 Da and VI was of 13725 Da. The tandem mass MS/MS analysis indicated that the PLA_2_s isolated corresponding to the fractions V-1, V-2, V-3 and VI were K49 PLA_2_ homologs, and V-4 was D49 PLA2 (Table 2).

**Table 2:** Protein identification results for *B. asper*-PLA_2_ by ESI MS/MS peptide sequence obtained from mass tandem MS/MS.

Fraction	MH^+^ (monoisotopic mass )	*z*	MS/MS-derived sequence	Data base ID	Species	Score		Reference
						Spectrum mill	Mascot	
P V-1	1944.87	3+	NPVTSYGAYGCNCGVLGR	Q9PVE3.1	*B. asper* M1-3-3	17.76	52	[35]
	1394.64	2+	TIVCGENNSCLK	AAF66702.1	*B. moojeni* Myotoxin II precursor	14.21	87	[36]
	460.74	2+	MILQETGK	Q9PRT7.1	*B. asper* Myotoxin IV	-	37	[37]
	434.05	2+	CCYVHK	AAF66702.1	*B. moojeni* Myotoxin II precursor	-	25	[36]
P V-2	1944.87	3+	NPVTSYGAYGCNCGVLGR	Q9PVE3.1	*B. asper* M1-3-3	12.65	68	[35]
	1394,57	2+	TIVCGENNSCLK	1CLP_B	*B. asper* Myotoxin II	-	53	[38]
	1637.76	3+	DKTIVCGENNSCLK	AAF66702.1	*B. moojeni* Myotoxin II precursor	12.23	24	[36]
	952.78	2+	ELCECDK	AAF66702.1	*B. moojeni* Myotoxin II precursor	-	27	[36]
	996.80	1+	ENLDTYNK	AAF66702.1	*B. moojeni* Myotoxin II precursor	12.69	31	[36]
	802.36	2+	AVAICLR	Q9PRT7.1	*B. asper* Myotoxin IV	-	36	[37]
P V-3	1944.87	3+	NPVTSYGAYGCNCGVLGR	Q9PVE3.1	*B. asper* M1-3-3	10.85	43	[35]
	1394.64	2+	TIVCGENNSCLK	AAF66702.1	*B. moojeni* Myotoxin II precursor	-	57	[36]
	1637.74	3+	DKTIVCGENNSCLK	AAF66702.1	*B. moojeni* Myotoxin II precursor	17.52	31	[36]
	952.78	2+	ELCECDK	AAF66702.1	*B. moojeni* Myotoxin II precursor	-	27	[36]
	802.36	2+	AVAICLR	Q9PRT7.1	*B. asper* Myotoxin IV	-	32	[37]
	1533.66	2+	SYGAYGCNCGVLGR	AAF66703.1	*B. neuwiedi pauloensis* PLA2 homolog	17.32	63	[39]
P V-4	2064.41	2+	DATDRCCFVHDCCYGK	P20474.2	*B. asper* Myotoxin III	9.51	30	[35]
	1728.75	2+	EICECDKAAAVCFR	1GMZ_A	*B. pirajai* Piratoxin III	8.61	-	[40]
	1506.59	2+	SGVIICCEGTPCEK	P20474.2	*B. asper* Myotoxin III	-	64	[35]
	862.56	2+	MILEETK	P20474.2	*B. asper* Myotoxin III	-	35	[35]
	794.57	2+	AAAVCFR	P86974.1	*B. leucurus* blD-PLA2	-	26	[41]
	1273.31	2+	YMAYPDLLCK	P20474.2	*B. asper* Myotoxin III	-	42	[35]
	675.45	2+	YSYSR	P20474.2	*B. asper* Myotoxin III	-	23	[35]
P VI	1329.72	2+	MILQETGKNPAK	Q9IAT9.2	*B. neuwiedi pauloensis* BnSP-7	11.63	42	[39]
	1533.66	2+	SYGAYGCNCGVLGR	AAF66703.1	*B. neuwiedi pauloensis* PLA_2_ homolog	17.92	52	[39]
	790.04	1+	LTGCNPK	P86453.1	*B. alternatus* BaTx	-	28	[42]
	1637.56	2+	DKTIVCGENNSCLK	AAF66702.1	*B. moojeni* Myotoxin II precursor	-	21	[36]
	1394.57	2+	TIVCGENNSCLK	1CLP_B	*B. asper* Myotoxin II	-	77	[38]

**Figure 3:**
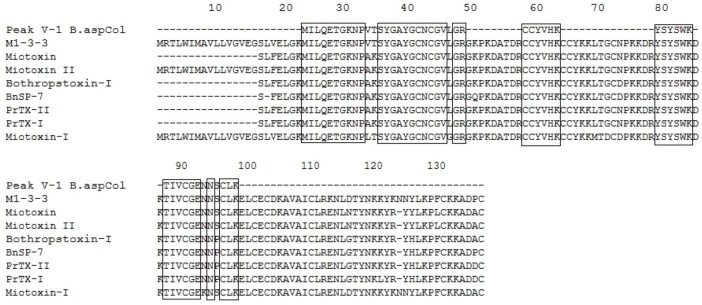
Multiple sequence alignment of Fraction P V-1. The boxes represent conserved amino acids. *B. asper* M1-3-3 Swiss Protein ID: Q9PVE3.1, GenBank ID: AAF14241.1|AF109911, Myotoxin *B. asper* PDB ID: 1CLP_A, Myotoxin-II *B. asper* Swiss Protein ID: P24605.3, Bothropstoxin-Ia *B. jararacussu* GenBank ID: CAA55334.2, BnSP-7 *B. neuwiedi* Q9IAT9.2, Piratoxin-II *Bothrops pirajai* P82287.1, Piratoxin-I *B. pirajai* Swiss Protein ID: 58399.2, Myotoxin-I *B. atrox* Swiss Protein ID: P82287.1.

**Figure 4:**
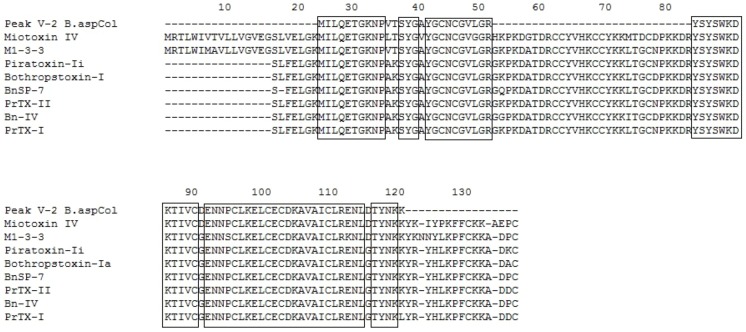
Multiple sequence alignment of Fraction P V-2. The boxes represent conserved amino acids. Myotoxin- IV *B. asper* Swiss Protein ID: P0C616, M1-3-3 *B. asper* Swiss Protein ID: SP|Q9PVE3.1, GenBank ID: AAF14241.1|AF109911, Piratoxin-Ii *B. pirajai* PDB ID: 2QLL_A, Bothropstoxin-Ia *B. jararacussu* GenBank ID: CAA55334.2, BnSP-7 *B. neuwiedi* Swiss Protein ID: Q9IAT9.2, Piratoxin-II *B. pirajai* Swiss Protein ID: P82287.1, BnIV *B. neuwiedi* PDB ID: 3MLM_A, Piratoxin-I *B. pirajai* Swiss Protein ID: 58399.2.

**Figure 5:**
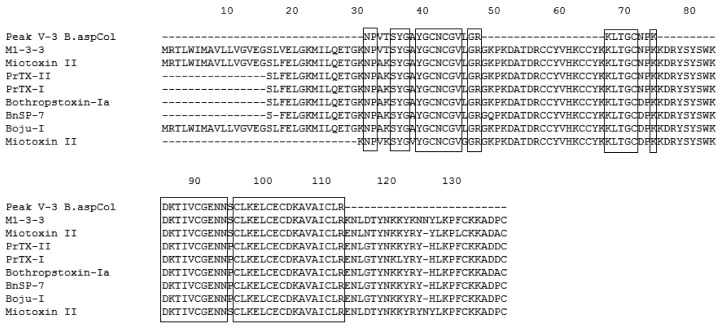
Multiple sequence alignment of Fraction P V-3. The boxes represent conserved amino acid. M1-3-3 *B. asper* Swiss Protein ID: Q9PVE3.1, Myotoxin-II *B. asper* Swiss Protein ID: P24605.3, piratoxin-II *B. pirajai* Swiss Protein ID: P82287, Piratoxin-I *B. pirajai* Swiss Protein ID: 58399.2, Bothropstoxin-Ia *B. jararacussu* GenBank ID: CAA55334.2, BnSP-7 *B. neuwiedi* Swiss Protein ID: Q9IAT9.2. BOJU-I *B. jararacussu* Swiss Protein ID: Q90249.3, Myotoxin-II *B. moojeni* GenBank ID: AAF66702.1.

**Figure 6:**
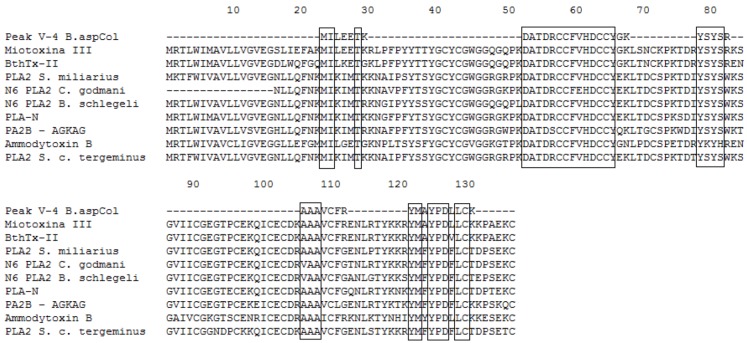
Multiple sequence alignment of Fraction P V-4. The boxes represent conserved amino acid. Myotoxin-III *B. asper* Swiss Protein ID: P20474.2, BthTx-II *B. jararacussu* Swiss Protein ID: P45881.1, PLA_2_ *S. miliarius* GenBank ID: ABY77926.1, N6 PLA_2_ *C. godmani* GenBank ID: AAR14161.1, N6 PLA_2_ *B. schlegelii* GenBank ID: AAR14162.1, PLA-N *T. flavoviridis* GenBank ID: BAC56893, PA2B_AGKAG *D. acutus* Swiss Protein ID: Q1ZY03, Variant ammodytoxin-B *V. aspis* GenBank ID: CAE47279.1, PLA_2_ *S. c. tergeminus* Accession number GenBank ID: ABY77930.1.

**Figure 7:**
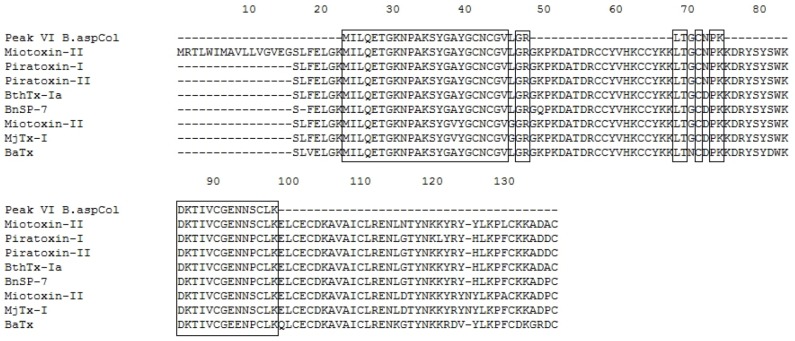
Multiple sequence alignment of Fraction P VI. The boxes represent conserved amino acid. Myotoxin-II *B. asper* Swiss Protein ID: P24605.1, Piratoxin-I *B. pirajai* Swiss Protein ID: P58399.2, Piratoxin-II *B. pirajai* Swiss Protein ID: P82287.3, BthTx-Ia *B. jararacussu* GenBank ID: CAA55334, BnSP-7 *B. neuwiedi* Swiss Protein ID: Q9IAT9.2, myotoxin-II *B. moojeni* PDB ID: 1XXS_2, MjTx-I *B. moojeni* Swiss Protein ID: P82114.1, BaTx *B. alternatus* Swiss Protein ID: P86453.1.

Additionally, the identified peptides were subjected to BLAST analysis to determine their identity with other phospholipases. The results confirmed the high identity of these peptides with PLA_2_s from the venoms of *B. asper, B. neuwiedi*, *B. jararacussu*, *B. pirajai* and *Cerrophidion godmani*, among others (see Figure 3, Figure 4, Figure 5, Figure 6, Figure 7).

The results of the alignments show that the PLA_2_s and PLA_2_ homologues purified from the venom of *B. asper* from Colombia are similar to other PLA_2_s and PLA_2_ homologues present in other *Bothrops* snakes. In addition, the PLA_2_ D49 shows homology with other PLA_2_s from *Bothrops*, being higher with those of *B. asper* from Costa Rica (see Figure 5).

### 2.6. Cytotoxic Activity

**Figure 8:**
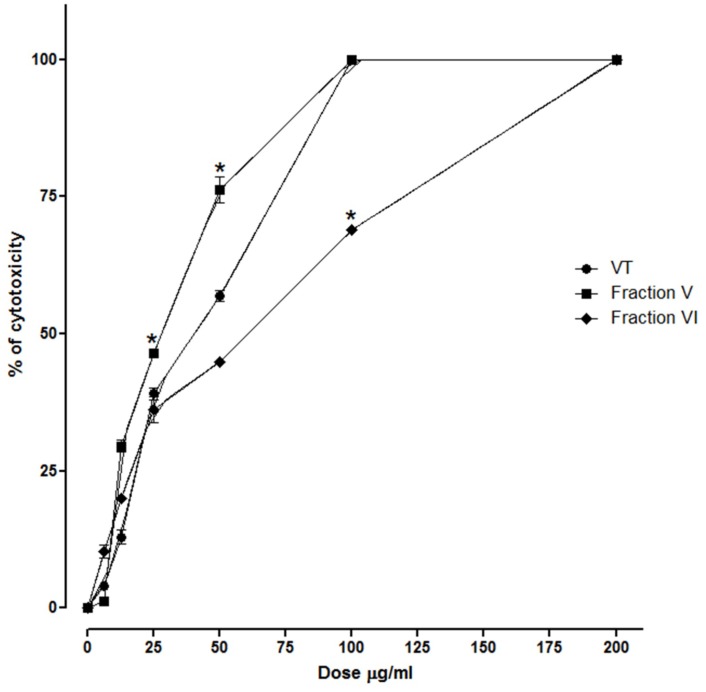
Cytotoxic activity of *B. asper* venom and isolated fractions on human peripheral blood mononuclear cells.VT venom, Fraction V, fraction VI. * *p* ≤ 0.05 compared to different doses.

Analysis of the cytotoxic effect of the whole venom and the different fractions tested showed that fraction V was more cytotoxic than whole venom or fraction VI on PBMC cells (see Figure 8).

The cytotoxic activity of venoms and PLA_2_s is a problem in using these in future biomedical applications. However, our results show that the PLA_2_ isolated exerts an antimalarial effect at a lower dose than that required to induce cytotoxicity in PBMC and indirect hemolysis.

Other authors have shown that cytotoxic activity is dependent on serum in suspensions of tumor cells and red blood cells [43]. In some experiments, we cultured cells with fetal bovine serum 2% (FBS) and inactivated serum or plasma, and in these conditions, the cytotoxic dose was still higher than the antimalarial dose (results not shown).

### 2.7. Acute Toxicity

The LD_50_ of the whole venom of *B. asper* was 3566 µg/kg (2561 to 3693), whereas no lethality was observed in mice injected with fractions V and VI at doses as high as 15,000 µg/kg (see Table 1). 

The envenoming of *B. asper* induces local and systemic symptoms, such as edema, pain and bleeding, among others, due to the effect of different toxins in the venom, such as PLA_2_, serine proteinases and metalloproteases, among others [19,44,45,46,47,48,49]. The low toxicity of fraction V and of the PLA_2_ homologue isolated from fraction VI compared with the venom indicates their low overall toxicity in mice and reinforces the concept that these fractions are good lead compounds in the search for antimalarial activity. This is in agreement with reports on the use of snake venom PLA_2_s to inhibit microorganisms, such as bacteria and fungi, as well as parasites including *Giardia duodenalis*, *Trypanosoma cruzi*, *Leishmania spp* and *P. falciparum* [17,30,31,50,51,52].

## 3. Materials and Methods

### 3.1. Venom and Reagents

The venom was obtained by manual milking of 40 adult specimens from different regions of Colombia held in captivity at the Serpentarium of the University of Antioquia (Medellín, Colombia). Once extracted and pooled, the venoms were centrifuged (3000 rpm, 15 min), and the resulting supernatants were lyophilized and stored at −20 °C until use.

Acetonitrile (CH_3_CN) and trifluoroacetic acid (CF_3_COOH) HPLC grade were purchased from Fisher Scientific (Loughborough, UK). Histopaque^®^-1077, RPMI-1640 medium culture, Thiazolyl Blue Tretrazolium Bromide (MTT) and dimethyl sulfoxide (DMSO) were purchased from Sigma (Sigma-Aldrich, St Louis, MO, USA). Water for HPLC was deionized to a degree of purity of 17 Ω.

### 3.2. Venom Fractionation

PLA_2s_ were purified from 50 mg of whole venom of *B. asper* dissolved in phosphate-buffered saline (PBS), pH 7.2, and passed through a CM-Sephadex C_25_ ion exchange column (1.8 × 120 cm) at the flow rate 1.0 mL/min on a low-pressure chromatography system (Econo-System, BioRad, Hercules, CA, USA). The resulting fractions were analyzed for their PLA_2_ activity and then PLA_2_ positive fractions submitted to a reverse phase HPLC (RP-HPLC) (Shimadzu, Model Prominence, Shimadzu Corporation, Kyoto, Japan) in a C_18_ column (pore 5 µm, 250 mm × 4.6 mm mark RESTEK Bellefonte, PA, USA) using a linear gradient (0%–100%) acetonitrile (*v*/*v*) in 0.1% (*v*/*v*) trifluoroacetic acid at a flow rate 1.0 mL/min. Finally, fractions were lyophilized and stored at −20 °C until use.

### 3.3. Electrophoresis and Molecular Mass Determination

Protein homogeneity of the obtained fractions were determined by electrophoresis under reducing and non-reducing conditions in SDS-polyacrylamide gel electrophoresis (SDS-PAGE) 15% [53]. Protein molecular weight was estimated according to a molecular weight markers range of 97.4 to 14.4 kDa (BioRad, Philadelphia, PA, USA). The gels were stained with Coomassie Brilliant Blue G-250. The molecular masses of PLA_2_ fractions were confirmed by direct-infusion mass spectrometry in an IonTrap (series 6310, Agilent Technologies, Santa Clara, CA, USA). 

### 3.4. Protein Iidentification by HPLC-nESI-MS/MS

The PLA_2_s and PLA_2_ homologues isolated from *B. asper* venom (fractions V and VI see results, Figure 1B,C) were digested in solution with trypsin (0.1 ng) at 30 °C (Agilent Technologies, Santa Clara, CA, USA) overnight, according to the manufacturer’s instructions, and injected onto a nano LC-ESI-MS/MS system (Agilent Technologies, Santa Clara, CA, USA) using a nano column C_18_ (Agilent Zorbax 300SB-C18, 150 × 0.075mm, 3.5 μm) coupled to a mass spectrometer IonTrap MSD series 6300 (Model 6310, Agilent Technologies, Santa Clara, CA, USA) [54]. MS/MS mass spectra were obtained in positive mode, dynamic range 200–1200 Da; Electrospray at 2 kV and 230 °C dry temperature, trap drive 200 ms. Charged state deconvolution of the MS/MS spectra were determined using the ChemStation G2070-91126 (Agilent Technologies, Santa Clara, CA, USA). 

### 3.5. Search Database

Deconvoluted profile spectra were used to search online the MASCOT [55] and Spectrum Mill (Agilent Technology, Santa Clara, CA, USA) in the NCBInr database for protein identification. The parameters of the search included digestion with trypsin and Carbamidomethyilation modified (C) as fixed modification. The minimum score for the intensity of each fraction was 50%, monoisotopic mass, mass tolerance of 2.5 Da and a way to search for identity.

### 3.6. BLAST Search of the Identified Peptides

The identified peptides were subjected to a BLAST search [56] to determine the homology with other PLA_2_ family proteins. This homology was performed in BLASTP, the search parameters being non-redundant protein sequence (nr) and a snake organism.

### 3.7. Acute Toxicity of the Venom and Fractions

The Median Lethal Dose (LD_50_) was determined by the Spearman-Karber method (World Health Organization, 1981) using groups of four mice (Swiss-Webster mice strain) injected intraperitoneally (IP) with varying doses of either fractions or whole *B. asper* venom, previously dissolved in 0.5 mL PBS, pH 7.2. Fatalities were recorded within 48 h, and the results were expressed as the average of three repetitions.

### 3.8. Cytotoxic Activity

Peripheral blood mononuclear cells (PBMC) were separated by centrifugation of citrated human blood (400*g*, 30 min) with Histopaque^®^-1077 (Sigma-Aldrich, St Louis, MO, USA), washed with PBS and transferred to 96 well plates at a concentration of 3 × 10^5^ cells/well. Cells were cultured with different concentrations of fractions (37 °C, 5% CO_2_) for 24 h. After this time, 40 µL of MTT was added and incubated for 3 h (same conditions as described). The reaction was halted by adding 130 µL of dimethyl sulfoxide (DMSO) and readings were performed in a microplate reader at 420 nm. The 50% cytotoxic dose was calculated by linear regression [57].

### 3.9. Indirect Hemolysis

This was evaluated following the method that uses agarose gel-erythrocyte-egg yolk [58,59]. We estimated the minimum indirect hemolytic dose (MIHD), defined as the dose of venom producing a hemolytic halo of 20 mm in diameter after 20 h. In addition, indirect hemolytic activity was assessed on red blood cells in suspension. For this, different doses of either the whole venom or fractions V and VI were incubated with fresh human red blood cells for 30 min at 37 °C in the presence of 250 µL of inactivated human serum, inactivated human plasma, egg yolk or PBS. Afterwards, samples were centrifuged, and the percentage of lysis was determined by recording the absorbance at 540 nm as an index of released hemoglobin. As a control of 100% hemolysis, 2%Triton X-100 was used. The results were expressed as percentage of lysis, and the venom or toxin concentration producing 100% hemolysis was determined.

### 3.10. Cultivation of *Plasmodium falciparum*

Based on the procedure described by Trager and Jensen [60], parasites were grown at 37 °C in A+ human erythrocytes to a hematocrit of 2% and 3%–6% parasitemia under an atmosphere of 3% CO_2_, 6% O_2_ and 91% N_2_. 

### 3.11. Determination of Percentage of Growth Inhibition of *P. falciparum* by *B. asper* PLA_2_ Fractions

Increasing concentrations of PLA_2_ fractions V and VI in complete medium were plated in 96-well plates (100 µL/well) and incubated with asynchronous *P. falciparum* FCB1 (1.5% parasitemia, 4% hematocrit, 100 µL/well). Parasites were incubated as previously described [60]. After 24 h, 0.5 mCi of ^3^H-hypoxanthine was added to the culture, and parasites were cultured for further 24 h at the same conditions. Finally, the plates were freeze-thawed, and parasites were harvested onto filter paper, added to liquid scintillation cocktail and the incorporation of ^3^H-hypoxanthine determined in a Microbeta counter 1450 (Wallac, Perkin Elmer, Waltham, MA, USA).

The percentage of growth inhibition was calculated based on 100% uptake of the 3H-hypoxanthine of controls (parasites in culture medium, incomplete RPMI). Growth inhibition was calculated based on 100% uptake of the 3H-hypoxanthine control in parasites in the absence of PLA_2_s or PLA_2_ homologues. The IC_50_ values correspond to the venom or toxin concentration required to kill 50% of the parasites within 48 h, and was determined from dose-response curves according to Desjardins *et al*. [58]. 

### 3.12. Statistical Analysis

The results are presented as mean ± S.E.M of three replicates, and experimental differences between means were determined by analysis of variance followed by Dunnett’s test for intragroup comparisons. Significance was set up at *p* < 0.05.

## 4. Conclusions

Our observations suggest that PLA_2_s and PLA_2_ homologues present in the venom of *Bothrops asper* represent promising lead compounds in the search for novel antimalarial agents. Further studies should be performed on the identification of the molecular determinants of this activity.
